# Treatment of localized extranodal NK/T cell lymphoma, nasal type: a systematic review

**DOI:** 10.1186/s13045-018-0687-0

**Published:** 2018-12-20

**Authors:** Seok Jin Kim, Sang Eun Yoon, Won Seog Kim

**Affiliations:** Division of Hematology and Oncology, Department of Medicine, Samsung Medical Center, Sungkyunkwan University School of Medicine, 50 Irwon-dong, Gangnam-gu, Seoul, 135-710 South Korea

**Keywords:** Extranodal NK/T cell lymphoma, Chemoradiotherapy, Localized disease

## Abstract

Extranodal natural killer/T cell lymphoma (ENKTL), nasal type, presents predominantly as a localized disease involving the nasal cavity and adjacent sites, and the treatment of localized nasal ENKTL is a major issue. However, given its rarity, there is no standard therapy based on randomized controlled trials and therefore a lack of consensus on the treatment of localized nasal ENKTL. Currently recommended treatments are based mainly on the results of phase II studies and retrospective analyses. Because the previous outcomes of anthracycline-containing chemotherapy were poor, non-anthracycline-based chemotherapy regimens, including etoposide and l-asparaginase, have been used mainly for patients with localized nasal ENKTL. Radiotherapy also has been used as a main component of treatment because it can produce a rapid response. Accordingly, the combined approach of non-anthracycline-based chemotherapy with radiotherapy is currently recommended as a first-line treatment for localized nasal ENKTL. This review summarizes the different approaches for the use of non-anthracycline-based chemotherapy with radiotherapy including concurrent, sequential, and sandwich chemoradiotherapy, which have been proposed as a first-line treatment for newly diagnosed patients with localized nasal ENKTL.

## Background

Extranodal natural killer/T cell lymphoma (ENKTL), nasal type, is a rare subtype of non-Hodgkin lymphoma [1]. ENKTL is characterized by the invariable infection of lymphoma cells with the Epstein–Barr virus (EBV), and the diagnostic term ENKTL originates from its immunophenotype and predominant extranodal presentation [2]. Most patients present with stage IE/IIE disease involving the nasal cavity and adjacent sites [3]. The treatment of localized nasal ENKTL is a major issue in the management of ENKTL [4]. However, given its rarity, there is a lack of consensus on the treatment of localized nasal ENKTL. The currently recommended treatments are mainly based on the results of phase II studies and retrospective analyses, and there is no standard therapy based on randomized controlled trials. In this article, we review the current recommendations for the treatment of localized ENKTL and the results of recent clinical studies relevant to the future management of localized ENKTL.

### Treatment for newly diagnosed patients with localized nasal ENKTL

In contrast to other lymphomas, the treatment outcome of anthracycline-containing chemotherapies, such as CHOP (cyclophosphamide, doxorubicin, vincristine, and prednisone), is poor in patients with ENKTL because the tumor cells express high concentrations of the multidrug-resistant P-glycoprotein, which results in resistance to anthracycline [5–7]. Therefore, currently, non-anthracycline-based chemotherapy regimens have been suggested as a first-line treatment for localized as well as advanced ENKTL. These non-anthracycline-based regimens include ifosfamide and methotrexate, which are not affected by P-glycoprotein, and etoposide, which is effective for treating EBV-associated lymphoproliferative disorders [8, 9]. l-Asparaginase is another main drug used for non-anthracycline-based chemotherapy because tumor cells cannot synthesize l-asparagine and die when their stores of l-asparagine are depleted by l-asparaginase [10]. l-Asparaginase-based regimens have outstanding response rates of more than 80% in patients with refractory or relapsed ENKTL [11, 12].

Radiotherapy is also used as an initial treatment for localized ENKTL, especially in cases involving the nasal cavity and adjacent sites, because the lesion frequently presents as a small mass confined to the nasal cavity and radiotherapy can produce a rapid response. Accordingly, the upfront use of radiotherapy has been reported to improve the local control rate in patients with localized ENKTL involving the nasal cavity [13–16]. However, radiotherapy alone is currently considered as insufficient for improving survival because a substantial number of patients experience local and systemic relapse after radiotherapy alone [17–19]. As a result, the combined approach of non-anthracycline-based chemotherapy with radiotherapy has been proposed as a first-line treatment for newly diagnosed patients with localized nasal ENKTL, as discussed below.

#### Concurrent chemoradiotherapy

**Fig. 1:**
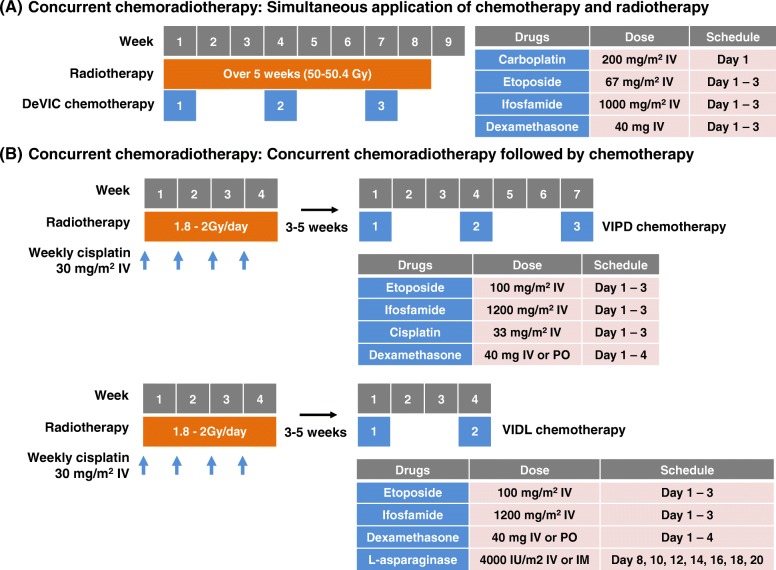
**a** In this type of concurrent chemoradiotherapy, systemic chemotherapy is overlapped with radiotherapy. **b** Radiotherapy is combined with weekly administration of cisplatin, and adjuvant chemotherapy is followed after the completion of concurrent chemoradiotherapy

Concurrent chemoradiotherapy is based on previous findings that the upfront use of radiotherapy improves the treatment outcome of localized ENKTL. In the DeVIC (dexamethasone, etoposide, ifosfamide, and carboplatin) regimen, radiotherapy (50 Gy) and three cycles of a two-thirds dose of DeVIC chemotherapy are initiated simultaneously (Fig. 1a). This regimen was reported to have a 77% complete response (CR) rate and 81% overall response rate [20]. The follow-up updated analysis reported that the 5-year overall survival (OS) and progression-free survival (PFS) rates were 70% and 63%, respectively, with acceptable late toxicities [21]. The benefit of this simultaneous application of radiotherapy and chemotherapy may include reduced risk of systemic progression during local therapy. However, as radiotherapy is overlapped with chemotherapy, the hematologic and non-hematologic toxicity may increase. Other regimens including ESHAP (etoposide, steroid, high-dose Ara-C and cisplatin) and DEP (dexamethasone, etoposide and cisplatin) were concurrently administered with radiotherapy, and they also showed high rates of hematologic toxicities (Table 1) [22, 23].

**Table 1:** Chemoradiotherapy regimens for stage IE/IIE nasal extranodal NK/T cell lymphoma

Regimen	Number	Drugs	RT	CR	G3/4 neutropenia	PFS
Concurrent chemoradiotherapy						
Simultaneous application of chemotherapy and radiotherapy						
DeVIC [20]	27	Dexamethasone, etoposide, ifosfamide, carboplatin	50 Gy	77%	90.9%	5-year 67%
ESHAP [22]	13	Etoposide, steroid, Ara-C, cisplatin	40 Gy	92%	92%	2-year 72%
DEP/DVIP [23]	33	Dexamethasone, etoposide, and cisplatin/dexamethasone, etoposide, ifosfamide, and cisplatin	50.4 Gy	63%	85%	5-year 60%
Weekly cisplatin with radiotherapy followed by chemotherapy						
VIPD [24]	30	Etoposide, ifosfamide, cisplatin, dexamethasone	40–52.8 Gy	80%	46.7%	3-year 85%
VIDL [25]	30	Etoposide, ifosfamide, dexamethasone, L-asparaginase	40–44 Gy	87%	80%	5-year 73%
MIDLE [26]	28	Methotrexate, ifosfamide, dexamethasone, L-asparaginase, etoposide	36–44 Gy	82%	91.3%	3-year 74%
GDP [27]	32	Gemcitabine, dexamethasone and cisplatin	56 Gy	84.4%	41%	3-year 84%
Sequential chemoradiotherapy						
SMILE [29]	17	Dexamethasone, methotrexate, ifosfamide, L-asparaginase, etoposide	> 40 Gy	69%	n.a.	n.a.
DICE-L [31]	33	Cisplatin, ifosfamide, etoposide, dexamethasone, L-asparaginase	45 Gy	90.9%	n.a.	5-year 89%
Sandwich chemoradiotherapy						
GELOX/PGEMOX [32]	27	Gemcitabine, L-asparaginase, oxaliplatin/pegaspargase, gemcitabine, oxaliplatin	56 Gy	74.1%	33.3%	2-year 86%
GELOXD/GEMOXD [35]	167	Gemcitabine, L-asparaginase, oxaliplatin, dexamethasone/pegaspargase, gemcitabine, oxaliplatin, dexamethasone	50 Gy	88.6%	23.4%**	3-year 72.8%

*RT* radiotherapy, *CR* complete response, *PFS* progression-free survival, *n.a* not applicable due to lack of data

**Grade 3/4 leukopenia for the whole group

Another concurrent chemoradiotherapy regimen includes radiotherapy with weekly cisplatin and adjunct chemotherapy (Fig. 1b). In the first report of this regimen, concurrent chemoradiotherapy was followed by three cycles of VIPD (etoposide, ifosfamide, cisplatin, and dexamethasone), and an 80% CR rate and 85% 3-year PFS rate were reported [24]. When cisplatin is used as a radiosensitizer, this approach can reduce the radiation dose by around 40 Gy. However, there is a potential risk of systemic disease progression during the period of radiotherapy. Subsequent phase II studies have tried different regimens with reduced cycle length and addition of l-asparaginase. Concurrent chemoradiotherapy followed by two cycles of VIDL (etoposide, ifosfamide, dexamethasone, and l-asparaginase) have been reported to have an 87% CR rate and 73% 5-year PFS rate [25]. Similar outcomes have been reported for the addition of triweekly l-asparaginase to concurrent chemoradiotherapy followed by two cycles of MIDLE (methotrexate, ifosfamide, dexamethasone, l-asparaginase, and etoposide), including an 82% CR rate and 74% 3-year PFS rate [26]. However, concurrent chemoradiotherapy followed by VIDL was less likely to cause febrile neutropenia than VIPD and MIDLE (Table 1). Similar outcomes have also been reported for other concurrent chemoradiotherapy regimens including radiotherapy with weekly cisplatin followed by 3 cycles of GDP (gemcitabine, dexamethasone, and cisplatin) in patients with localized nasal ENKTL (Table 1) [27].

#### Sequential chemoradiotherapy

**Fig. 2:**
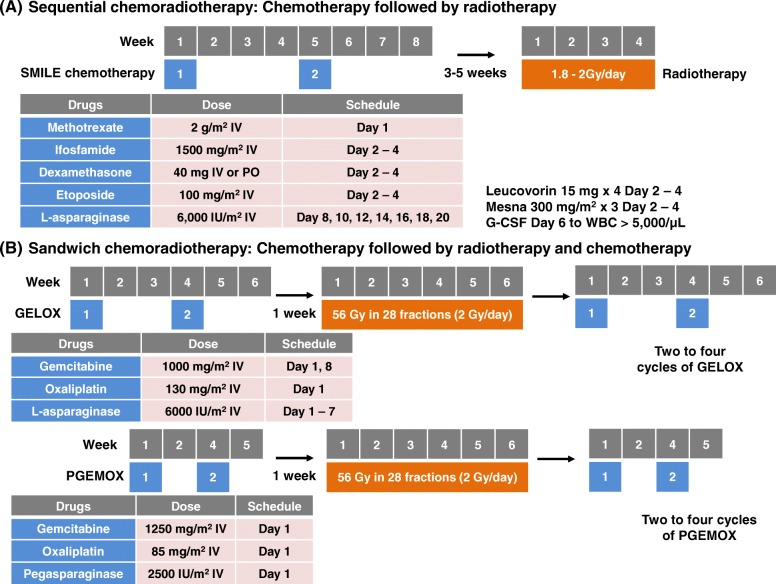
**a** Chemotherapy is followed by radiotherapy. **b** Chemotherapy is followed by radiotherapy and additional cycles of chemotherapy

The efficacy of SMILE (dexamethasone, methotrexate, ifosfamide, l-asparaginase, and etoposide) chemotherapy was demonstrated in a phase II study of patients with stage IV ENKTL [28]. SMILE chemotherapy is used widely as a standard of care for patients with advanced ENKTL. The Asia Lymphoma Study Group analyzed the outcome of patients with localized nasal ENKTL who received SMILE followed by radiotherapy in clinical practice. This group reported a 69% CR rate and 90% overall response rate [29]. The sequential treatment with 2–4 cycles of SMILE and radiotherapy may be recommended as a treatment option for patients with localized nasal ENKTL (Fig. 2a) [30]. However, the hematologic toxicity was severe and common in patients receiving SMILE chemotherapy. Therefore, the SMILE regimen should be used cautiously in elderly and frail patients. Other regimens used for sequential chemoradiotherapy, including DICE-l (cisplatin, ifosfamide, etoposide, dexamethasone, and l-asparaginase), have similar outcomes (Table 1) [31].

#### Sandwich chemoradiotherapy

Sandwich chemoradiotherapy comprises sequential chemoradiotherapy followed by additional chemotherapy (Fig. 2b). In a phase II study, patients with localized nasal ENKTL initially received at least two cycles of GELOX (gemcitabine, l-asparaginase, and oxaliplatin) and then received radiotherapy (56 Gy). Within 1 week after completion of radiotherapy, they received GELOX for 2–4 cycles, giving a maximum total of six cycles of GELOX. For patients who experienced hypersensitivity to L-asparaginase, pegaspargase was used instead of l-asparaginase (PGEMOX regimen: pegaspargase, gemcitabine, and oxaliplatin). This approach achieved a 74% CR rate and 96% overall response rate [32]. The updated analysis after the long-term follow-up reported an 85% 5-year OS rate and 74% 5-year PFS rate [33]. The benefit of sandwich chemoradiotherapy may be the reduction in the intensity of chemotherapy. The main toxicities were grades 1 and 2, and no treatment-related deaths occurred during GELOX or PGEMOX [32]. Therefore, this regimen has been shown to have promising outcomes with a manageable toxicity profile in elderly patients with localized nasal ENKTL [34]. A recent retrospective analysis from three Chinese hospitals also showed a 3-year PFS of 72.8% with manageable grade 3/4 leucopenia (23.4%) [35]. However, this approach might require a longer duration of treatment compared with concurrent or sequential chemoradiotherapy.

#### Optimal dose and technique of radiotherapy

As mentioned above, radiotherapy can be used in concurrent, sequential, or sandwich chemoradiotherapy for localized nasal ENKTL. However, the optimal dose and techniques of radiotherapy for the treatment of localized nasal ENKTL has not been elucidated. A previous Chinese retrospective study showed radiotherapy of 50 Gy could be effective for patients with low tumor burden such as stage I and normal LDH [36]. On the other hand, the safety and efficacy of concurrent chemoradiotherapy with 40 Gy were reported by a Korean retrospective study [37]. Thus, acute toxicities were tolerable during the concurrent chemoradiotherapy, and grade ≥ 3 toxicity was found in only 4.8% of patients. Although 30% of patients experienced grade 2 nausea and mucositis during the treatment period, no case of grade ≥ 2 late complication was observed with a median follow-up of 56 months [37]. Thus, for primary radiotherapy, a dose of 50 Gy could be considered whereas a radiation dose could be reduced to 40 Gy in concurrent chemoradiotherapy with cisplatin administration. In addition to radiation dose, as radiation technique has improved, the mode of radiotherapy also might be an important factor for treatment outcome. Although there are no controlled trials comparing 3-dimension conformal radiotherapy (3D-CRT) with intensity-modulated radiotherapy (IMRT), IMRT has significantly lower toxicities than 3D-CRT. Thus, IMRT was recommended for patients with localized nasal ENKTL by the International Lymphoma Radiation Oncology Group [38]. Recently, a retrospective analysis with 1691 Chinese patients with localized ENKTL demonstrated better 5-year OS and PFS in patients receiving IMRT (75.9% and 67.6%) than patients with 3D-CRT (68.9% and 58.2%, *p* < 0.05). This survival benefit of IMRT was also significant in patients receiving combined treatment with L-asparaginase- or gemcitabine-containing chemotherapy [39]. Thus, IMRT could be an effective radiation technique for localized nasal ENKTL in terms of survival outcome and toxicity profiles.

### Risk-adapted treatment approach for localized nasal ENKTL

Not all patients with localized nasal ENKTL have a better treatment outcome than do those with advanced disease because some patients can develop early relapse, which has a dismal prognosis despite the initial presentation as localized ENKTL. By contrast, other patients may have a truly localized disease with a small tumor burden. Therefore, identification of patients at high risk of treatment failure may help in developing the risk-adapted treatment approach in patients with localized nasal ENKTL. Currently, the prognostic index of natural killer lymphoma (PINK), which includes age > 60 years, stage III or IV disease, distant lymph node involvement, and nonnasal disease, is used as a prognostic model for patients with ENKTL [40]. The PINK model is particularly useful in identifying those patients who should be treated for advanced disease. However, only a small proportion of patients with localized nasal ENKTL might belong to high risk according to the PINK model. Therefore, a modified PINK model that includes the EBV DNA titer in the blood at the time of diagnosis (PINK-E) may be more useful for identifying patients at risk of treatment failure among those with localized nasal ENKTL. The EBV DNA titer in blood has been suggested as a potential surrogate marker for disease activity because of its significant association with a high tumor burden and poor treatment outcomes [41–43]. Moreover, the presence of circulating EBV DNA at the end of treatment correlates with the risk of relapse, which suggests its potential as a marker of residual disease [44].

Recent studies comparing the outcomes of concurrent, sequential, and sandwich chemoradiotherapy have shown similar efficacy, although these were based on the results of retrospective and indirect comparisons [45, 46]. The long-term outcomes of patients receiving concurrent chemoradiotherapy regimens including DeVIC and VIDL in Japan and Korea were also similar [47]. Therefore, all these approaches may be recommended as a first-line treatment for localized nasal ENKTL. However, the patient’s age, comorbidity, and risk of systemic progression can influence the physician’s choice of first-line treatment. For example, if a patient is expected to have a high risk of systemic progression such as high titer of EBV DNA, the physician might consider starting intensified chemotherapy, such as SMILE, and sequential radiotherapy.

### Treatment of elderly patients with localized nasal ENKTL

**Fig. 3:**
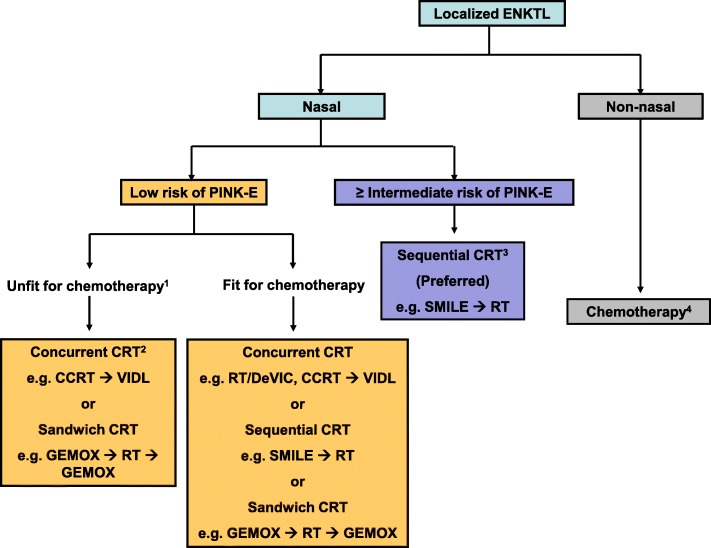
Treatment recommendation for localized NK/T cell lymphoma. ^1^Elderly or frail patients with poor performance. ^2^Only concurrent chemoradiotherapy (radiotherapy with weekly cisplatin) without adjunct chemotherapy can be tried for patients unfit for chemotherapy especially if a patient has a small mass. ^3^Intensified systemic chemotherapy can be a preferred option for patients at high risk of treatment failure. ^4^Non-nasal type should be treated like advanced disease. *CRT* chemoradiotherapy, CCRT concurrent chemoradiotherapy

The treatment strategy should be different for elderly patients, as those of other hematological malignancies because comorbidities and diminished organ function are frequent in elderly patients [48]. Indeed, a previous Chinses study analyzing elderly patients with localized nasal ENKTL receiving radiotherapy alone or a combination treatment of CHOP or CHOP-like chemotherapy with radiotherapy have shown poor 5-year OS and PFS, 42% and 40%, respectively [49]. However, our single-center analysis with 51 patients (≥ 60 years) demonstrated the outcome of elderly patients with localized nasal ENKTL was better than that of advanced disease [50]. In particular, concurrent chemoradiotherapy with cisplatin followed by l-asparaginase-containing chemotherapy such as VIDL was tolerable and most patients completed the planned treatment without treatment-related mortality [50]. Favorable survival outcome was also reported in elderly patients with localized ENKTL receiving GELOX/PGEMOX followed by radiotherapy [34]. Accordingly, a recent multicenter study from the China reported a favorable curability of 321 elderly patients with localized ENKTL [51]. Non-anthracycline-based chemotherapy combined with radiotherapy significantly improved 5-year PFS compared to anthracycline-based chemoradiotherapy (71.2% vs. 44.2%, *p* = 0.017). Thus, an elderly patient might receive greater benefit from concurrent chemoradiotherapy followed by VIDL or sandwich chemoradiotherapy with GELOX/PGEMOX (Fig. 3). In addition, only concurrent chemoradiotherapy (radiotherapy with weekly cisplatin) without adjunct chemotherapy could be tried for patients unfit for chemotherapy especially if a patient has a small mass because radiotherapy with 50 Gy alone showed a favorable survival equivalent to the general population in elderly patients with low tumor burden such as stage I, normal LDH, and absence of primary tumor invasion [51].

### CNS prophylaxis for localized nasal ENKTL

Central nervous system (CNS) relapse is problematic because it can lead to poor prognosis in most patients with non-Hodgkin lymphoma. Because localized ENKTL frequently affects the nasal cavity and paranasal area near the CNS, ENKTL may increase the risk of CNS relapse. Our previous retrospective analysis showed that a small number of patients experienced CNS relapse (5.76%, 12/208) [52]. Most patients with CNS relapse had advanced disease but not localized disease involving the nasal cavity. Therefore, CNS evaluation and prophylaxis at the time of diagnosis may not be routinely indicated for patients with localized nasal ENKTL. However, the best method to identify patients with localized nasal ENKTL at risk of CNS relapse remains unclear.

### Treatment of localized nonnasal ENKTL

ENKTL can also involve other extranodal sites such as the skin, testis, intestine, and muscle [53], but it is not clear how these forms are biologically different from nasal ENKTL [54]. However, the prognosis of ENKTL involving nonnasal sites has been reported to be worse even in patients with localized disease [55, 56]. Initial radiotherapy may not produce a favorable outcome in patients with nonnasal disease because the disease can rapidly disseminate and progress, especially when it initially presents with high blood EBV titer. Therefore, patients with localized nonnasal ENKTL should be treated with systemic chemotherapy as used in the treatment of systemic disorders.

#### Treatment of relapsed patients with localized nasal ENKTL

Although the survival outcome of localized nasal ENKTL has improved, a substantial number of patients still relapse even after they achieve a CR [57]. Although there is no consensus on the salvage treatment for relapsed ENKTL, various treatment options might be applicable according to the pattern of relapse. For example, if patients relapse locally confined to nasal cavity without systemic symptoms and the time to relapse is longer than at least 2 years, retreatment with concurrent or sequential or sandwich chemoradiotherapy may be tried, as for newly diagnosed patients. However, if disease relapse occurs within 2 years after the completion of first-line treatment, systemic chemotherapy should be considered because the outcome of early disease relapse was found to be poor in a recent retrospective study of patients with localized nasal ENKTL receiving concurrent chemoradiotherapy [47].

## Conclusions

Patients with localized nasal ENKL should be treated with a combined chemotherapy and radiotherapy treatment. The physician may select the treatment approach from concurrent, sequential, or sandwich chemoradiotherapy regiments according to the patient’s status and risk. However, it is unknown which approach is the best for patients with localized nasal ENKTL. The risk of relapse and treatment-related toxicity remains a problematic issue for the use of the current regimens. Further studies are needed to develop more effective and tolerable treatment approaches for patients with localized nasal ENKTL.

## Abbreviations

3D-CRT: 3 dimension conformal radiotherapy
CHOP: Cyclophosphamide, doxorubicin, vincristine, prednisone
CNS: Central nervous system
CR: Complete response
DeVIC: Dexamethasone, etoposide, ifosfamide, carboplatin
DICE-l: Cisplatin, ifosfamide, etoposide, dexamethasone, l-asparaginase
EBV: Epstein–Barr virus
ENKTL: Extranodal natural killer/T cell lymphoma
GDP: Gemcitabine, dexamethasone, and cisplatin
GELOX: Gemcitabine, l-asparaginase, oxaliplatin
IMRT: Intensity-modulated radiotherapy
MIDLE: Methotrexate, ifosfamide, dexamethasone, l-asparaginase, etoposide
OS: Overall survival
PFS: Progression-free survival
PGEMOX: Pegaspargase, gemcitabine, oxaliplatin
PINK: Prognostic index of natural killer lymphoma
SMILE: Dexamethasone, methotrexate, ifosfamide, l-asparaginase, etoposide
VIDL: Etoposide, ifosfamide, dexamethasone, l-asparaginase
VIPD: Etoposide, ifosfamide, cisplatin, dexamethasone
